# Hyperlipidemia in tendon injury: chronicles of low-density lipoproteins

**DOI:** 10.1007/s00441-023-03748-8

**Published:** 2023-02-04

**Authors:** William H. Fang, Victor Bonavida, Devendra K. Agrawal, Finosh G. Thankam

**Affiliations:** Department of Translational Research, Western University of Health Sciences, Pomona, CA 91766 USA

**Keywords:** ECM disorganization, Hyperlipidemia, LDL, oxLDL, Rotator cuff tendon injury

## Abstract

Hyperlipidemia impacts millions of people globally and has been the major risk factor for developing atherosclerosis and cardiovascular disease. Interestingly, hyperlipidemic subjects exhibit increased incidence of rotator cuff tendon injury (RCTI) and disorganization of tendon matrix. Low-density lipoproteins (LDL) and its oxidized form (ox-LDL) play a crucial role in hyperlipidemia-driven pro-inflammatory responses in multiple tissues including the tendon. The signaling of oxLDL upregulates the inflammatory cytokines, chemokines, adhesion molecules, and the activation of monocytes/macrophages/resident tendon cells and matrix metalloproteinases impairing the tendon homeostasis resulting in the alteration of extracellular matrix. In addition, the hyperlipidemia-driven immune response and subsequent oxidative stress promote degenerative responses in the tendon tissue. However, the pathological mechanisms underlying the occurrence of RCTI in hyperlipidemia and the effect of ox-LDL in tendon matrix are currently unknown. The present review focuses on the implications and perspectives of LDL/oxLDL on the increased incidence of RCTI.

## Introduction

Hyperlipidemia is a chronic condition that describes various genetics and acquired disorders leading to elevated lipid levels in the body. Hyperlipidemia results in the increased risk of cardiovascular disease (CVD) as evident from the increased incidence at an early age (Balakumar 2016; Karr 2017). The advancements in medical sciences have benefitted the better management of hyperlipidemia and associated diseases. Moreover, the association of hyperlipidemia with musculoskeletal and tendon pathology has been documented (Yang et al. 2019). A seminal study reported that the patients with tendinopathy displayed increased level of circulatory cholesterol, LDL, and triglyceride with a concomitant reduction in HDL level suggesting the relationship between the two pathologies (Tilley et al. 2015); however, the underlying molecular mechanisms are obscure. Additionally, significant impairments in tendon integrity, structure, and biochemical makeup have been observed in patients with hyperlipidemia suggesting the increased prevalence of tendon injury or rupture (Soslowsky and Fryhofer 2016). Despite the clinical information, the pathological relationship between hyperlipidemia and tendon injuries is poorly understood at the molecular level. On this juncture, this review focuses on the cellular and biochemical mediators involved in the comorbidity of hyperlipidemia and rotator cuff tendon injury (RCTI) with an emphasis on LDL signaling.

## Rotator cuff tendon injury (RCTI)

RCTI refers to musculoskeletal injuries relating to the coexistence of degeneration and inflammation in the rotator cuff (RC) tendon. Clinically, RCTI is presented as pain, inflammation, and inability of the shoulder to perform various ranges of motion especially elevation and external rotation (Lewis et al. 2015). Hence, RCTI has been the major musculoskeletal complaint in both clinical and sports medicine (Cools et al. 2015). Additional contributing factors, including smoking, age, and genetics, have been associated with the incidence of RCTI (Lädermann et al. 2015), (Li and Hua 2016). Biochemically, the RC tendons are composed of mostly water and collagen molecules embedded in the extracellular matrix (ECM) (Woo et al. 2008). In the ECM of RC tendon, the collagen is assembled into a hierarchical structure starting with fibrils that form fibers that associate with fascicles, and the bundles of fascicles constitute the fascicular matrix (Thorpe et al. 2015). Despite multiple cell types in the tendon tissues, the majority of the tendon is constituted of tenocytes, tenoblasts, and tendon stem/stromal cells (TSCs) (Kannus 2000). Roughly, 95% of the dry matter in the tendon tissue is type I collagen with varying amounts of collagen types III, V, XI, XII, and XIV (Table 1) (Screen et al. 2015; Thorpe et al. 2013). The type I collagen (Col-I) fibrils display stiff structures that provide mechanical durability and strength to the tendon tissue (Thankam et al. 2018a). Additionally, type II collagen (Col-II) only exists in small quantities which is typically concentrated near the tendon-bone insertion points (Kumagai et al. 1994). Moreover, the type III collagen (Col-III) fibrils are crucial in healing process and are upregulated following tendon injury. Hence, the presence of Col-III indicates an abnormal/healing response, resulting in elastic, loosely organized tissue (Maffulli et al. 2000). Importantly, the increased ratio of Col-III/Col-I in the tendon reflects degenerated tendon contributing to decreased mechanical resistance in RCTI (D'hondt et al. 2018).

**Table 1:** Function and properties of collagen phenotypes

**Type**	**Function**	**Properties**
Collagen I	Structural Support in connective tissue, muscle, tendon, bone, and skin	Most abundant protein in all vertebrates. Assembles into fibers that form mechanical and structural scaffold
Collagen II	Primarily found in cartilage, the nucleus pulposus, and vitreous humor of the eye	Does not form fibrils, allows cartilage to entrap proteoglycan aggregates as well as provide tensile strength to tissue
Collagen III	Found in the skin, lungs, intestinal walls, and walls of blood vessels. Upregulated in healing and following injury	Composed of 3 alpha chains and can accommodate the expansion and contraction of tissues such as blood vessels and viscera. Indication of tendon damage, involved in the initial phase of healing, and significantly affects the tendon biomechanics
Collagen V	Contributes to the bone matrix, corneal stroma, and intestinal matrix of internal organs	Shown to regulate fibrillogenesis by nucleating collagen fibril formation; essential for the assembly of collagen I containing fibrils
Collagen XI	Associated with collagen II containing tissues, and expressed broadly during development of Collagen I tissues	Regulate fibrillogenesis by maintaining the spacing and diameter of type II collagen fibrils, and a nucleator for the fibrillogenesis of collagen types I and II
Collagen XII	Localized in the tendon matrix and associated with tenocytes in developing, maturing, and mature tendons	Regulates organization and mechanical properties of collagen fibril bundles in dense connective tissues and bone
Collagen XIV	Fibril associated collagen found mainly in skin, tendon, cornea, and articular cartilage	Regulates fibrillogenesis by limiting fibril diameter through prevention of lateral fusion of adjacent fibrils

## Hyperlipidemia

Hyperlipidemia, obesity, and higher BMIs have been associated with disorganized collagen fibrils in the tendon tissue (Taş et al. 2017). The increased density of adipocytes in hyperlipidemia results in the increased pool of proinflammatory signals, hormones, and pathological mediators which influence multiple organs including the RC tendon (Bray et al. 2017). A seminal study involving 49,914 subjects reported the strong association of hyperlipidemia/obesity and the incidence, progression, and complications of RCTI compared to the control subjects (Macchi et al. 2020). Another study highlights that the patients with BMI > 75 reflected morphologically different tendon patterns of ECM organization compared to the subjects with lower BMI (Steinberg et al. 2020). In a recent study, it was demonstrated in a cohort of 5856 individuals that hypercholesterolemia (defined as total cholesterol greater than 5 mmol/L) increased risk of tendon injury in the upper extremities by 1.5-fold, and metabolic syndrome increased by 2.5-fold (Skovgaard et al. 2021). Moreover, influence of the changes in life style following the hyperlipidemic burden and subsequent statin therapy on tendon homeostasis is largely unknown warranting further research.

### Low density lipoprotein

LDL is a complex lipoprotein containing various lipid species including triglycerides, phospholipids, and free/esterified cholesterol (Khosravi et al. 2018). Elevated levels of these plasma lipoproteins have been correlated with higher incidence of RCTI (Bolam et al. 2021; Longo et al. 2010). There are multiple pathways that promote the formation of oxidized LDL (oxLDL) which leads to increased levels of inflammatory cytokines, chemokines and adhesion molecules, activation of monocytes/macrophages and matrix metalloproteinase (MMPs), and upregulation of scavenger receptors (Steinberg 2009). Importantly, the subendothelial retention of native LDL and oxLDL triggers the infiltration and activation of inflammatory resulting in foam cell formation and the progression of atherosclerotic lesions (Ishigaki et al. 2009).

**Fig. 1:**
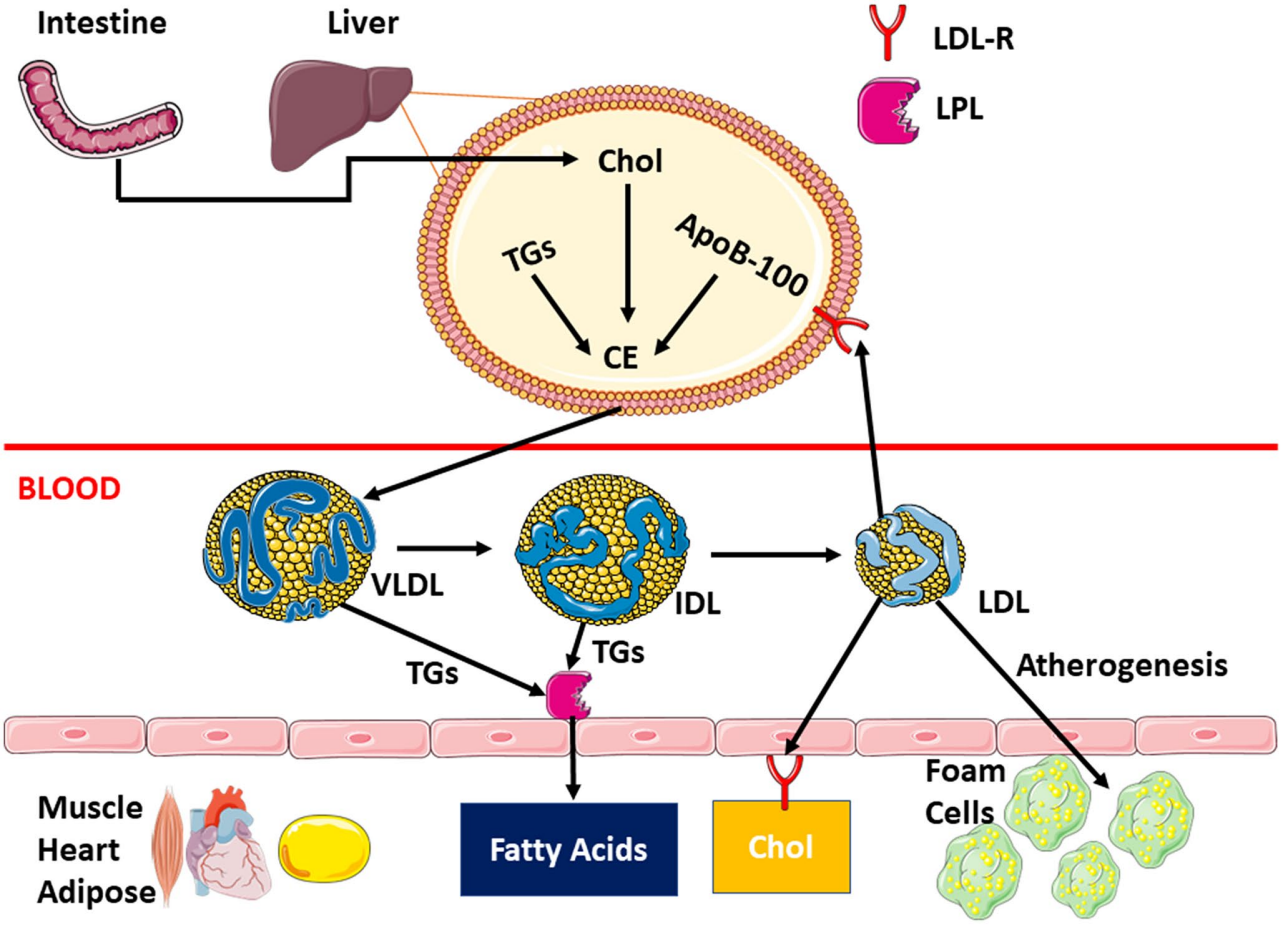
LDL molecular formation pathway from precursor lipid species. The molecular events involve binding of LDL particle on the plasma membrane, followed by internalization, and routing to the lysosome for degradation into fatty acids. However, an overabundance of LDL leads to atherogenesis through the formation of foam cells, lipid-laden macrophages, that localize on blood vessel walls

LDL particles circulate in the blood before binding to cell surface LDL receptors (LDL-R) which in turn mediate the cellular uptake of lipid components (Fig. 1) (Herz et al. 2000). The molecular events involve the binding of LDL particle on the plasma membrane, followed by internalization, and routing to the lysosome for degradation through a process called receptor-mediated endocytosis. Other family members in this receptor class include LDL-R, LDL receptor-related protein (LRP), megalin, very low-density lipoprotein receptors (VLDL-R), and ApoE receptor 2 (apoER2). The binding of structurally dissimilar ligands to these receptors results in the internalization in similar pathway (May et al. 2007). These receptors require Ca^2+^ for ligand binding.

The LDL-R is a cell surface glycoprotein comprised of 839 amino acids (Yamamoto et al. 1984) which is synthesized as an immature protein, processed in the Golgi apparatus, before turning into a mature form that is transported to the cell surface. LDL-R has an N-terminal ligand-binding domain consisting of seven cysteine rich complement type repeats. Additionally, it also contains 3 EGF precursor homology domains and one O-linked glycosylated domain (Hussain 2001). The entire receptor is rich in hydrophobic amino acid residues that promote membrane anchoring of cholesterol, while cytoplasmic membrane FDNPXY sequence is necessary for targeting of receptors. Studies have shown that the various repeats of negatively charged domains are necessary for a high binding affinity of cholesterol molecules; however, there is certain specificity that precludes all negatively (Goldstein et al. 1979). Specifically, the receptor binds apolipoprotein B (apoB) in LDL particles before being internalized by endocytosis via clathrin-coated pits involving the LDL receptor adaptor protein 1 (LDLRAP1) (Sniderman et al. 2010). After being endocytosed, the LDL molecule is degraded in an acidic lysosomal compartment and the cell receives LDL-derived cholesterol. This type of cholesterol further blocks sterol regulated membrane-bound transcription factors called sterol regulatory element-binding proteins (SREBPs). In cholesterol-depleted cells, SREBPs are normally synthesized in the endoplasmic reticulum, transported to the Golgi apparatus for processing, which activates the transcription of genes encoding HMG-CoA reductase (HMGCR) and the other enzymes of cholesterol biosynthesis and LDL receptor (Goldstein and Brown 2009). However, LDL-derived cholesterol blocks the transport to the Golgi, preventing the activation of HMGCR, to downregulate cholesterol synthesis, preventing cholesterol overload.

Low intracellular cholesterol levels trigger the activation of SREBPS to increase transcriptional activation of HMGCR (the rate limiting enzyme of cholesterol biosynthesis) and the downstream enzymes of the mevalonate (MVA) pathway (Sakakura et al. 2001). However, once the cell receives LDL-derived cholesterol, the transcription of the HMGCR gene is downregulated through the inactivation of the SREBP pathway via a feedback regulation. Additionally, the cholesterol content of the cell is regulated by the action of esterifying enzyme, acyl CoA:cholesterol acyltransferase (ACAT) and facilitating the storage (Kristiana et al. 2008).

The hyperlipidemia induces xanthomas (deposits of cholesterol in peripheral tissues) and accelerated atherosclerosis which increases risks of coronary heart disease. Xanthomas present as subcutaneous nodules with normal overlying skin and commonly occur on tendons, especially Achilles (Yang et al. 2019), tendon attachments, ligaments, fascia, and periosteum (Bell and Shreenath 2020). Mostly, the xanthomas appear in yellow color due to the presence of carotene found in lipids (Al Jasmi et al. 2019). Xanthomas cause pain, especially if localized on larger tendons, and lead to tendon rupture and weakness. Xanthomas bear cholesterol-rich materials and foams cells resulting from hyperlipidemia (Bath et al. 2010). There are rare cases of rotator cuff xanthomas reported; however, most of the pathology is found on the Achilles tendon. Hyperlipidemia normally leads to rotator cuff tears rather than xanthomas.

Native lipids (lipoproteins) do not induce foam cell formation; instead, the factors including the pathogenic modification of high local concentrations of lipids in connective tissue, the presence of qualitatively different lipoproteins at normal plasma lipid concentration, and increased extravasation of lipids, as well as dysfunction of the reverse cholesterol transport, are the key triggers (Zak et al. 2014). Typically, free cholesterol inhibits its de novo synthesis and the synthesis of LDL receptors. However, when phagocytic cells depend on the scavenger receptors [SR-A, SR-B1, CD36, lectin-like oxidized LDL receptor-1 (LOX-1)] for the uptake of oxidatively modified LDL particles, OxLDL (PrabhuDas et al. 2017). Interestingly, the oxLDL particles fail to activate the feedback receptors leading to a hyper-cholesterol environment, mediating the formation of xanthomas. Importantly, a positive relationship between the size of Achilles tendon xanthomas with titers of antibodies against OxLDL has been identified (Tsouli et al. 2005). Additionally, the decreased HDL concentration and disorders in the reverse cholesterol transport triggered the Achilles xanthomas despite normolipidemic subjects (Matsuura et al. 2005). Higher amounts of serum LDL have also been suggested to be responsible for the thickness of Achilles tendon, as well as RC tendinopathy, affecting the mechanical properties of the tendon and leading to higher rates of tendon injury (Scheel et al. 2004).

## OxLDL

LDL undergoes biochemical modifications especially oxidation in the sub-endothelial space of the vascular wall. Oxidation of LDL is catalyzed by multiple reactions including a lipoxygenase reaction (Kühn et al. 1994; Sigal et al. 1994), metal mediated oxidation (Ehrenwald et al. 1994; Lynch and Frei 1993; Sakurai et al. 1991; Sniderman et al. 2010), and peroxidase catalyzed reactions (Napoli et al. 1991). LDL oxidation is a complex process where both the apolipoprotein B100 and lipids present in LDL are modified. Reactive oxygen species (ROS) induce fragmentation of apoB, producing peptides of varying sizes from 14 to 500 kDa as well as protein carbonyls (Matsuura et al. 2008). Additionally, lipids and fatty acids such as cholesteryl esters, phospholipids, and triglycerides present in LDL are susceptible to oxidation by ROS releasing free and esterified fatty acid peroxides, aldehydes, and ketones that are further oxidized to amplify the damage. The formation of these products or the changes in the properties of circulating LDL are not guaranteed during the oxidation of LDL as many are secondary products of oxidation and their formation largely depends on the type of oxidant, the extent of oxidation, and the presence or absence of other agents such as redox metals (Parthasarathy et al. 2010).

In blood vessels, oxLDL activates endothelial cells by triggering the expression of adhesion molecules (Obermayer et al. 2018) which mediate the rolling and adhesion of circulating leukocytes (monocytes and T cells) to the endothelium. Following adhesion, the monocytes migrate into the intimal layer in response to chemokines and subsequently differentiate into macrophages that upregulate both toll-like receptors (TLRs) and scavenger receptors (SRs) (Pirillo et al. 2013). Additionally, stimulation of membrane bound CD36, TLR2, TLR4, and TLR6 results in the upregulation of pro-inflammatory mediators and initiate immune activation of macrophages and microglia (Chávez-Sánchez et al. 2010; Stewart et al. 2010). As the consequence of the macrophage activation, proinflammatory cytokines are released, ROS are synthesized, and proteolytic enzymes are secreted contributing to the matrix degradation. However, activation of TLRs in tendinopathies is still under debate. However, TLR expression contributes minimally to Achilles tendon degeneration, but triggers degradative tissue reactions (de Mos et al. 2009). In contrast, our group reported that under an oxidative environment (oxidative stress) there is increased level of the major damage associated molecular patterns (DAMPS), high mobility group box (HMGB1), which in turn maintains RAGE, TLR4, and TLR2 in an activated state (Thankam et al. 2018b). In patients with RCTI and glenoid arthritis, HMGB1 was significantly upregulated contributing to the pro-inflammatory responses aggravating the injury. Additionally, the increased levels of HMGB1 and RAGE in patients with superior labral anterior to posterior (SLAP) tears are most likely due to the ischemia/necrosis-mediated sterile inflammation associated with the injury (Thankam et al. 2016). Interestingly, these DAMP mediators have been upregulated in the tendon tissues of RCTI rat model which were progressively downregulated during the course of healing (Thankam et al. 2018b).

**Fig. 2:**
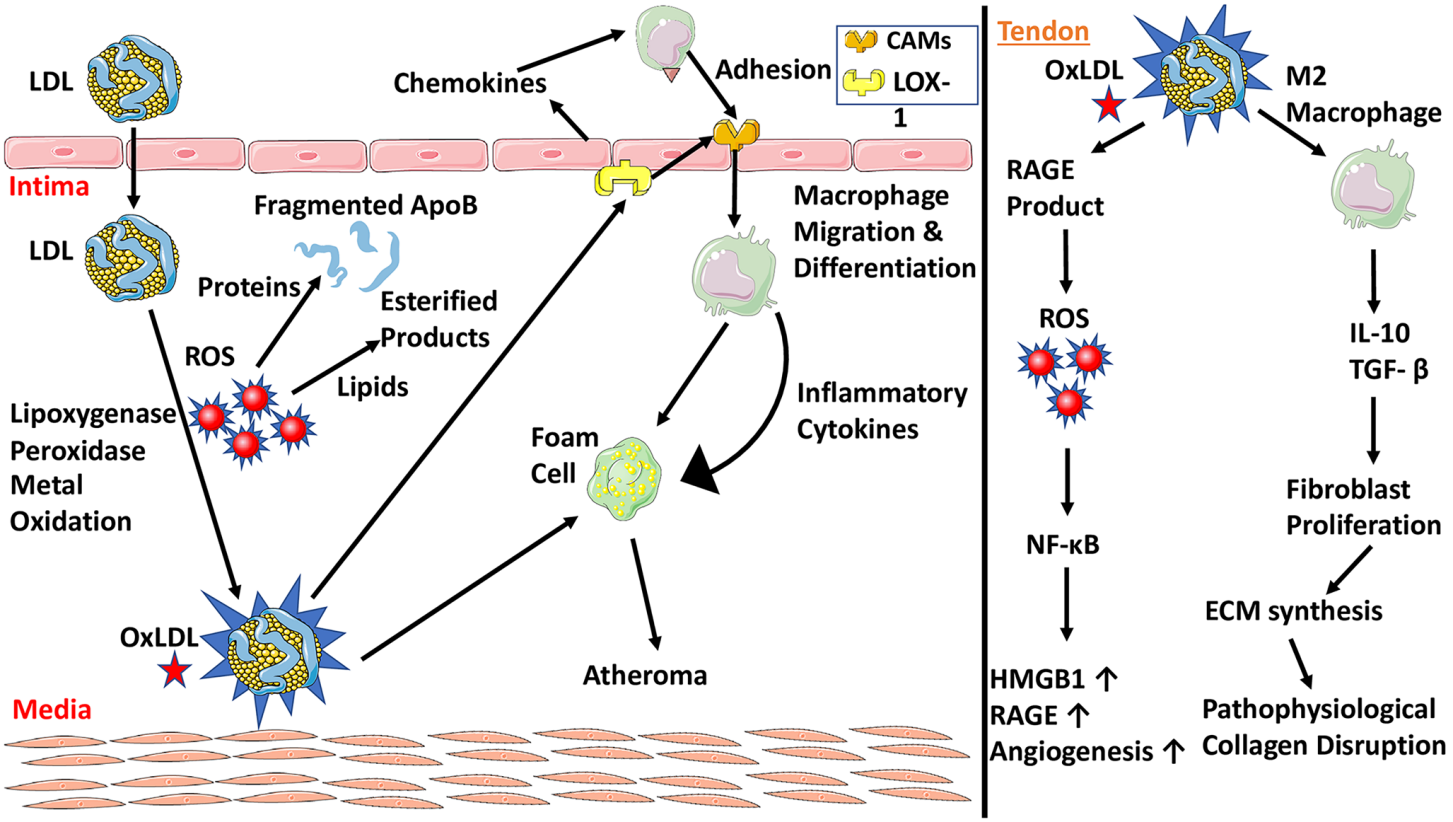
(Left panel) Oxidative molecules and free radicals lead to OxLDL formation. OxLDL increases inflammatory cytokines and polarizes macrophages to M2 phenotype. (Right panel) Downstream signaling of OxLDL and M2 leading to collagen disruption and angiogenesis

Importantly, oxLDL has been identified as a ligand for receptors for advanced glycation end products (RAGE) in hyperlipidemic conditions (Sun et al. 2009). RAGE accelerates the lipid deposition and foam cell formation in smooth muscle cells by increasing the uptake of oxLDL, and the production of ROS with subsequent activation of NF-kB. NF-kB upregulates a battery of inflammatory mediators leading to chronic inflammatory state (Farmer and Kennedy 2009). Logically, this inflammatory pathway could be the link to explain tendinopathies caused by these molecules. Evidently, harvested human bicep tendons displayed significantly increased expression of HMGB1, RAGE, and angiogenesis in RCTI patients (Thankam et al. 2016). It has been hypothesized that these molecules contribute to increased cell migration and angiogenesis, resulting in the recruitment of additional inflammatory cells and release of mediators promoting ECM disorganization and eventual tendinosis (Fig. 2).

Additionally, oxLDL stimulates the polarization of macrophages to M2 phenotype, leading to increased IL-10 and transforming growth factor beta (TGF-β) (Rios et al. 2013). TGF-β precipitates fibrotic disorders leading to tendon injuries and stimulates the migration and proliferation of fibroblasts and ECM synthesis (Stone et al. 2016). Chronic elevation of TGF-β induces definitive changes in tendon ECM and tendon fibroblasts contributing to altered cellular responses to healing. However, TGF-β1 expression is known to be variable in diseased human tendons, depending on the anatomy of the tendon and its disease stage (tendinopathy or tear) (Fenwick et al. 2001; Goodier et al. 2016). Overall, the oxLDL causes significant pathophysiological tendon alterations, leading to a degradative phenotype.

## Cholesterol in RCTI

**Fig. 3:**
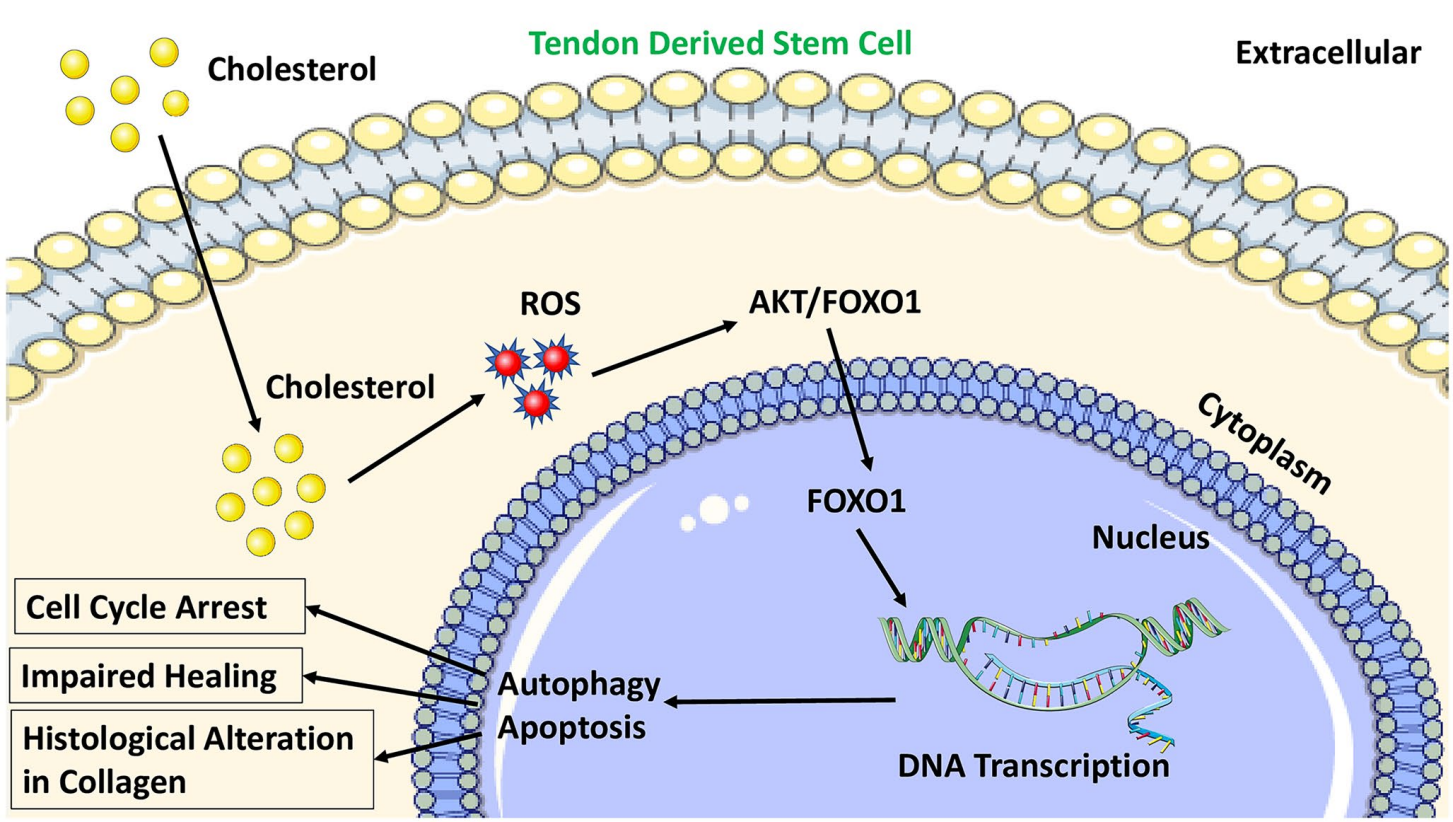
The intracellular macrophage activation leading to the increased production of proinflammatory cytokines and ROS. Cholesterol reacts with ROS to activate AKT/FOXO1 signaling leading to the histological alterations including degenerative damage in the tendon

Information from the animal models have suggested that higher serum concentrations of cholesterol favor the greater incidence of tendon pathology. High cholesterol-driven apoptosis and autophagy of Achilles tendon derived stem cells (TDSCs) through reactive oxygen species (ROS)-activated AKT/FOXO1 signaling have been reported suggesting the degenerative changes (Li et al. 2020) (Fig. 3). Moreover, cholesterol inhibits the proliferation and migration of tendon derived stem cells (TDSC) and induces cell cycle arrest. In a seminal study, the exposure of tendons to differing levels of cholesterol for varying amounts of time reduced the expression of Ki76, a proliferation marker, confirming an increase in cell cycle arrest (Beason et al. 2014) as evident from an increased proportion of G0/G1 phase cells and fewer G2/M and S phase cells. In addition, the increased cholesterol level significantly downregulated the expression of tendon cell markers with a simultaneous increase in ROS signaling via NF‐κB mediated pathway. As a result, significant histological alteration was evident in the experimental models with hypercholesterolemia (Li et al. 2019). Overall, high cholesterol inhibits tendon-related gene expression in TDSCs implying that pathogenesis in tendon injury is related to the alterations in tendon specific gene expression.

Apolipoprotein E (ApoE), the component of HDL, has been associated with its effects of alleviating pre-existing atherosclerotic lesions (Tangirala et al. 1999) as well as reducing LDL levels by promoting LDLR-dependent hepatic clearance of triglyceride-rich lipoproteins from the circulation (Rosenfeld et al. 1993). Interestingly, ApoE has been hypothesized to be protective for tendon function; however, the underlying mechanism is unknown. A recent study using ApoE knockout mice model reported that the consumption of a high-fat diet led to marked increase in oxLDL deposition in the load-bearing ECM of the tendon where the effect was aggravated in ApoE knockout group. Additionally, the lack of ApoE resulted in the increased pool of oxLDL and subsequent upregulation of matrix metalloproteinase 2 (MMP2) (Grewal et al. 2014). Similarly, another study reported an increase in the tendon stiffness and modulus in the supraspinatus tendons of hypercholesterolemic, ApoE knock-out mice, compared to control mice (Beason et al. 2011). Interestingly, a handful of reports concluded the impact of hypercholesterolemia in the pathogenesis of tendon injury. A summary of these studies is displayed in Table 2.

**Table 2:** Studies detailing the impact of hyperlipidemia in tendon injury

**Study model/patients**	**Method**	**Result**	**Reference**
Isolated Tendon-derived stem cells (TDSCs) from female rats	Analyzed levels of apoptosis in TDSCs after exposure to high levels of cholesterol for 24 h	Study indicated that high cholesterol induced apoptosis and autophagy through ROS-activated AKT/FOXO1 signaling in TDSCs, providing new insights into the mechanism of hypercholesterolemia-induced tendinopathy	(Li et al. 2020)
*N* = 64 Sprague Dawley rats, 32 received high cholesterol diet	Analyzed supraspinatus tendon biomechanical and histological evaluation	Supraspinatus Tendon healing and stiffness was decreased at 4 weeks and hypercholesterolemia can contribute to tendon injury and ability to heal after injury	(Beason et al. 2014)
*N* = 12 SD rats, 6 with intact ApoE and 6 with ApoE knockout	Fed high fat level diet until 10 months old. Achilles’ tendons were harvested and analyzed with H&E staining, immunostaining	Results revealed that tenocytes had changed morphologically, similar presentation to those in tendinopathy. High cholesterol may inhibit tendon related genes via ROS-activated NF-kB signaling	(Li et al. 2019)
*N* = 65 ApoE knockout mice	Analyzed histology, immunohistochemistry, cross-sectional area, RNA, and Ox LDL	Results showed no differences except in OxLDL deposition in tendons. This increase in OxLDL induced an upregulation of Mmp2 gene, which alters tendon structure by down regulating normal collagen genes	(Grewal et al. 2014)
*N* = 80; 40 male controls and 40 males deficient for ApoE representing high cholesterol	40, 14-week-old, and 40, 10-month-old mice groups. Each split into 20 control and 20 ApoE. Elastic Modulus and healing assessments were measured of the patellar tendons	Results showed patellar tendon elastic modulus in aging, or long-term exposure, to hypercholesterolemia was significantly reduced which leads to detrimental effects on tendon mechanics	(Beason et al. 2011)
*N* = 205	Divided into 3 groups: Normal LDL group, borderline LDL, and hypercholesterolemia group. Achilles’ tendon thickness (ATT) was measured via radiograph	ATT was markedly higher in the borderline group than in the normal group (*P* < .05) and the ATT was significantly higher than the normal group (*P* < .005). Positive correlation between LDL-C levels and ATT (*P* < .001)	(Wang et al. 2018)
*N* = 47	Analyzed blood samples for total cholesterol, triglycerides (TG), LDL and HDL in those who underwent Achilles’ tendon repair (ATR)	Was found to have significant differences between ATR and control group in TC, LDL-C, and TG values. It was found that patients who underwent repair had significant increases in concentrations of TC, TGs, and LDL-C	(Ozgurtas et al. 2003)
*N* = 287	Retrospectively looked at the group of patients who underwent Achilles’ tendon repair surgery	Was found that those patients who underwent Achilles’ tendon repair had associated increased levels in cholesterol, triglycerides, and LDL-C	(Yang et al. 2020)
*N* = 51	2 groups according to LDL levels; *N* = 24 in hyperlipidemia group; 27 in non-hyperlipidemia Patellar tendon and rectus femoris muscle shear velocities were measured	Found that blood LDL levels, independent of BMI, had an impact on tendon stiffness. There was a positive moderate statistically significant correlation between LDL and patellar tendon shear wave velocity. Faster the shear wave velocity, stiffer the tendon potentially leading to increased predisposition for injury	(Torgutalp et al. 2020)
N = 2612 over 17 studies	Systematic and Metanalysis Review; inclusion criteria based on Lipid levels, use of lipid lowering drugs and tendinopathy	Results showed people had altered tendon structure or tendon pain in those with significantly higher cholesterol, LDL, and TGs	(Tilley et al. 2015)
*N* = 36	16 participants with FH (10 men 6 women); 16 healthy ones with matched BMI and gender	FH patients had significantly decreased stiffness compared to controls with lower threshold of hysteresis due to different Achilles loading patterns. Decreased stiffness can be possible be linked to different loading rate, rather than an increase in tendon strain. Concluding that increased cholesterol and lipid levels alter tendon structure and predispose tendinous injury	(Squier et al. 2021)
*N* = 1208 familial hypercholesterolemia patients	Analyzed genetic variants in LDL oxidation pathways	Higher number of risk alleles were found in the oxLDL pathway in association with presence of tendon xanthomas	(Oosterveer et al. 2010)
*N* = 14 heterozygous FH patients	Measured Achilles tendon diameter on sonography, total RNA pools and also intracellular lipid contents	It was shown that macrophages in patients with Achilles xanthomas present had differential gene expression in the presence of oxLDL, proposing that xanthoma formation in tendons is associated with increased levels of lipids and oxLDL	(Artieda, et al. 2005)
*N* = 80 patients with heterozygous FH	Measured levels of oxLDL, autoantibodies against oxLDL and LDL associated Phospholipase A2	Antibodies formed against oxLDL are independently associated with Achilles tendon thickness suggesting that they may play a role in Achilles tendon xanthomas in FH patients	(Tsouli et al. 2006)
Literature review	Compiled articles discussing the pathogenesis, detection and treatment	Achilles tendon is the most common site for xanthoma formation. LDL is deposited into the tissues where it is converted into oxLDL by macrophages within tendon tissue	(Tsouli et al. 2005)

Abboud et al. (Abboud and Kim 2010) discovered that total cholesterol, triglycerides, and LDL-C levels were higher in patients with RCTI where HDL-C levels were lower than the control. In contrast, another study reported no significant difference between serum triglycerides and total serum cholesterol in RCTI (Longo et al. 2010). Also, a retrospective study found that patients prescribed for Achilles’ tendon repair surgery displayed increased levels of total cholesterol, LDL-C, and triglycerides with significant lipid deposits in tendon tissue compared to that of the healthy people (Wang et al. 2018). In addition, total cholesterol and LDL-C levels were significantly higher, while HDL-C levels were lower in patients with Achilles’ tendon repair than compared to controls (Ozgurtas et al. 2003). In a cross-sectional case-controlled study, patients with hyperlipidemia displayed significantly higher shear wave velocities in the patellar tendon indicating that the high levels of LDL impaired the biomechanical properties (Torgutalp et al. 2020). Interestingly, the LDL level had a direct impact on patellar tendon stiffness independently of body mass index. Similarly, hyperlipidemia and cholesterol accumulation were the major contributing factors to Achilles’ tendon injury in patients with familial hypercholesterolemia and tendinous xanthomas (Squier et al. 2021).

Importantly, the xanthoma formation has been associated with higher intracellular lipid content and higher inflammatory responses of macrophages to oxLDL. Therefore, xanthoma formation aggravates the risk of tendon inflammation due to abnormal inflammatory response and formation of macrophages (Artieda et al. 2005). Antibodies against oxLDL have been correlated with Achilles’ tendon thickness, and the patients demonstrated abnormal Achilles’ tendon echo structure with higher levels of oxLDL suggesting that immune cells from patients with familial hypercholesterolemia have increased predisposition to forming foam cells in the presence of oxLDL (Tsouli et al. 2006). In addition, there is significant association between moderate and high perioperative total cholesterol and LDL levels and the rates of repeat surgery after primary arthroscopic rotator cuff repair (Cancienne et al. 2017; Werner et al. 2017). This suggests that these elevated lipids also lead to rotator cuff pathological changes; however, further research is needed in this area. Overarchingly, the LDL levels correlate with the levels of oxLDL which eventually turn into tendinous xanthomas altering gross structure of the tendons. The intimate relationship between LDL and oxLDL and xanthomas cause tendons to increase in thickness and the thickness of the tendon returns closer to baseline following lowered levels of LDL (indirectly oxLDL) (Tsouli et al. 2005).

In the ApoE knockout mice group with the setting of a high fat diet, there was a significant increase in oxLDL levels in the load bearing parts of the tendon ECM. The ApoE knockout mice exhibited drastic decrease in tendon function and downregulation of Col IA1 genes compared to controls with increased MMP2 (Grewal et al. 2014).

## Summary

Hyperlipidemia significantly alters the pathophysiology of tendon tissues; however, the exact underlying mechanisms are unclear. This critical review provides insights into potential link between the increased concentration of LDL and oxLDL in the bloodstream and tendon injury with an emphasis on RCTI. OxLDL signaling upregulates the inflammatory cytokines, chemokines, and adhesion molecules as well as activation of monocytes/macrophages and MMPs impairing the tendon homeostasis resulting in the alteration of ECM. In addition, the activated macrophages upregulate both toll-like receptors (TLRs) and scavenger receptors (SRs) leading to ROS generation and thus promoting degenerative responses. Furthermore, the upregulation of DAMPs including HMGB1 in hyperlipidemia and the downstream signaling enhances the overall pool of pro-inflammatory signals facilitating the aggravated co-morbidity of RCTI. Additionally, RAGE accelerates the lipid deposition and foam cell formation by increasing the uptake of oxLDL and the production of ROS with subsequent activation of NK-kB. NK-kB upregulates inflammatory mediators leading to chronic inflammatory state, leading to increased incidence of tendinopathies especially RCTI. Also, oxLDL stimulates the polarization of macrophages to M2 phenotype, leading to increased expression of IL-10 and TGF-β resulting in fibrotic disorders in the tendon. Overall, the hyperlipidemia affects almost every part of the body including the tendons; however, the information regarding the underlying molecular pathology is limited warranting further research. Moreover, the elucidation of the underlying molecular mechanisms and the targets to intervene could open multiple translational opportunities for the development of novel therapeutic strategies in the management of RCTI.
